# Circulating Wnt Signaling Inhibitors and Osteoprotegerin in Women with Newly Diagnosed Overt Thyroid Dysfunction

**DOI:** 10.3390/metabo16050308

**Published:** 2026-04-30

**Authors:** Mariya Zhivkova Miteva, Maria Mitkova Orbetzova, Boyan Ivanov Nonchev, Delyana Miteva Davcheva, Kostadin Gigov

**Affiliations:** 1 Department of Endocrinology and Metabolic Diseases, Faculty of Medicine, Medical University of Plovdiv, 4002 Plovdiv, Bulgaria; maria.orbetzova@mu-plovdiv.bg (M.M.O.); nonchev_md@abv.bg (B.I.N.); 2 Clinic of Endocrinology and Metabolic Diseases, “St. George” University Hospital, 4000 Plovdiv, Bulgaria; 3 Clinic of Endocrinology and Metabolic Diseases, “Kaspela” University Hospital, 4001 Plovdiv, Bulgaria; 4 Department of Clinical Laboratory, Faculty of Medicine, “St. George” University Hospital, Medical University of Plovdiv, 4000 Plovdiv, Bulgaria; delyana.davcheva@mu-plovdiv.bg; 5 Section of Plastic Reconstructive and Aesthetic Surgery and Thermal Trauma, Department of Propedeutics of Surgical Diseases, Faculty of Medicine, “St. George” University Hospital Plovdiv, Medical University of Plovdiv, 4002 Plovdiv, Bulgaria; kostadin.gigov@phd.mu-plovdiv.bg

**Keywords:** autoimmune thyroid disorders, bone metabolism, osteoblast activity, Wnt pathway inhibitors

## Abstract

**Highlights:**

**What are the main findings?**

- DKK-1, SOST, and OPG levels are altered in women with newly diagnosed overt thyroid dysfunction.
- Correlations between these markers, thyroid hormones, and autoantibodies suggest modulation of bone metabolism even before measurable bone loss.

**What are the implications of the main findings?**

- Combined assessment of DKK-1, SOST, and OPG may help detect early biochemical changes in bone metabolism in overt thyroid disease.
- These biomarkers could guide monitoring and risk stratification prior to detectable decreases in bone mineral density.

**Abstract:**

**Background**: Thyroid hormones influence bone metabolism, and autoimmune thyroid diseases may further impact skeletal homeostasis. Wnt signaling inhibitors, including Dickkopf-1 (DKK-1) and sclerostin (SOST), as well as osteoprotegerin (OPG), play key roles in regulating bone formation and resorption. This study aimed to evaluate circulating DKK-1, SOST, and OPG in women with newly diagnosed overt thyroid dysfunction. **Methods**: This cross-sectional study included 62 women with newly diagnosed, untreated overt thyroid dysfunction (35 hypothyroid and 27 hyperthyroid) and 33 age- and BMI-matched healthy controls. Serum levels of DKK-1, sclerostin, and OPG were measured using ELISA. Thyroid function and autoantibodies were assessed using automated immunoassays. Correlation analysis was performed to evaluate associations between variables. **Results**: Serum DKK-1 levels were significantly elevated in both hypothyroid and hyperthyroid women compared with controls (*p* < 0.001). Sclerostin levels showed a non-significant trend toward higher values. OPG levels were significantly increased in hyperthyroid patients and moderately elevated in hypothyroid patients. Significant positive correlations were observed between OPG and FT3 (r = 0.42, *p* = 0.001) and FT4 (r = 0.43, *p* = 0.001). In hypothyroid patients, OPG correlated positively with TgAb (r = 0.46, *p* = 0.007). A strong positive correlation was found between DKK-1 and SOST (*p* < 0.001), while DKK-1 was negatively associated with age (*p* < 0.05). **Conclusions**: Overt thyroid dysfunction is associated with significant alterations in circulating Wnt signaling inhibitors and OPG. These findings suggest a potential role of Wnt signaling and immune–bone interactions in thyroid-related changes in bone metabolism.

## 1. Introduction

Thyroid hormones play a critical role in skeletal development and bone maintenance, influencing both osteoblast and osteoclast activity [1,2]. Overt thyroid dysfunction, whether hypo- or hyperthyroidism, has been associated with alterations in bone turnover markers and increased fracture risk [3,4]. Autoimmune thyroid diseases (AITD), such as Hashimoto’s thyroiditis and Graves’ disease, not only affect thyroid hormone levels but also induce immune-mediated effects that may impact bone metabolism [5,6].

The Wnt/β-catenin signaling pathway is essential for osteoblast differentiation and bone formation, while its inhibitors, including Dickkopf-1 (DKK-1) and sclerostin (SOST), negatively regulate bone formation [7,8]. These proteins are primarily produced by osteocytes and play a key role in the fine regulation of bone remodeling. Osteoprotegerin (OPG), a soluble decoy receptor for RANKL, regulates osteoclast differentiation and protects against excessive bone resorption [9,10,11,12].

Previous studies suggest that circulating levels of Wnt inhibitors and OPG may reflect early changes in bone metabolism in patients with thyroid dysfunction [13,14,15]. However, available data remain limited and somewhat inconsistent, particularly regarding the combined assessment of DKK-1, SOST, and OPG in women with newly diagnosed overt thyroid disease.

Based on the known effects of thyroid hormones on bone remodeling and the regulatory role of Wnt signaling in osteoblast function, we hypothesized that circulating levels of Wnt pathway inhibitors (DKK-1 and SOST) and OPG are altered in women with overt thyroid dysfunction, reflecting early changes in bone metabolism prior to measurable alterations in bone mineral density.

In this study, we evaluated serum levels of SOST, DKK-1, and OPG in women with newly diagnosed overt hypothyroidism and hyperthyroidism compared with healthy controls, with the aim of elucidating their potential involvement in thyroid hormone-related changes in bone remodeling.

## 2. Materials and Methods

### 2.1. Study Design

This was a cross-sectional observational study investigating circulating levels of Wnt signaling inhibitors and OPG in women with overt thyroid dysfunction.

### 2.2. Study Population and Ethics

The study included 62 women with newly diagnosed, untreated, autoimmune thyroid dysfunction, subdivided into hypothyroid (*n* = 35) and hyperthyroid (*n* = 27) groups. Diagnoses were based on clinical evaluation and laboratory assessment of thyroid function and autoimmunity.

All patients included in the study had overt (clinical) thyroid dysfunction, defined by abnormal serum TSH and free thyroid hormone levels (FT4 and/or FT3) accompanied by clinical symptoms of hypo- or hyperthyroidism. Patients with subclinical thyroid dysfunction were excluded.

A control group of 33 clinically healthy women was included. Controls were selected to match patients in terms of age, body mass index (BMI), and menopausal status. The mean age and BMI did not differ significantly between patients and controls (*p* > 0.05). The distribution of menopausal status was comparable between groups (60,4% premenopausal, 39.6% postmenopausal).

Inclusion criteria:

1. Women older than 20 years.
2. Newly diagnosed autoimmune thyroid dysfunction (clinical hypothyroidism or hyperthyroidism).
3. Signed written informed consent.

Exclusion criteria:

1. Current treatment with levothyroxine or antithyroid drugs.
2. Use of oral contraceptives or hormone replacement therapy in the last 6 months.
3. Use of calcium-phosphate supplements, vitamin D, or other medications affecting calcium-phosphorus metabolism in the last 6 months.
4. Coexisting diseases affecting bone metabolism (severe systemic or endocrine disorders, immunological or infectious diseases, malignancies).
5. Current pregnancy or lactation.

Ethics statement: The study was approved by the Scientific Ethics Committee of the Council for Scientific Research at Medical University—Plovdiv (protocol № P-1502/06.06.2019). All participants provided written informed consent prior to inclusion in the study.

Clinical examinations were conducted at the Department of Endocrinology and Metabolic Diseases, University Hospital “St. George”, Plovdiv, Bulgaria. All laboratory analyses were performed at the Central Clinical Laboratory of the same hospital, following routine internal quality control procedures using manufacturer-provided control materials.

### 2.3. Laboratory Methods

#### 2.3.1. Sample Collection

Fasting venous blood samples were collected between 7:00 and 9:00 a.m. using a closed-system blood collection device. Serum was separated from cells within 30 min of venipuncture by centrifugation at 3000 rpm for 15 min using a horizontal rotor. Aliquots were either analyzed immediately or stored at −20 °C. Samples showing hemolysis, lipemia, or icterus were excluded.

#### 2.3.2. Hormonal and Antibody Assays

Serum thyroid function and autoimmunity markers were measured using automated immunoassays on the Access 2^®^ analyzer (Beckman Coulter, Brea, CA, USA) with original reagents.

- TSH—Two-step sandwich immunoenzymatic assay; reference range 0.34–5.60 μIU/mL.
- FT4—CLIA; reference range 7.86–14.41 pmol/L.
- FT3—Two-step competitive immunoenzymatic assay; reference range 3.8–6.0 pmol/L.
- TPOAb—Two-step sandwich immunoenzymatic assay; reference range ≤ 9 IU/mL.
- TgAb—Two-step sandwich immunoenzymatic assay; reference range < 4.0 IU/mL.
- TRAb—ELISA method (Multiskan reader with dedicated software and Wellwash 4 MK2 washer (Fisher Scientific, Hampton, NH, USA); sensitivity 1 IU/L).

These assays were performed to confirm thyroid dysfunction and characterize the study population. TRAb was measured to identify patients with potential Graves’ disease; however, all analyses of bone markers were conducted according to thyroid function status (hypo- or hyperthyroid), without further subdivision by autoantibody profile.

#### 2.3.3. Measurement of Wnt Signaling Inhibitors and OPG

Serum DKK-1 and SOST were measured using ELISA kits (MyBioSource, San Diego, CA, USA), and OPG using an ELISA kit (Bender MedSystems, Vienna, Austria), according to manufacturers’ protocols. Polyclonal antibodies specific for each analyte were used. Color development was read at 450 nm, with intensity directly proportional to analyte concentration. Values are expressed in pg/mL. All samples were analyzed in duplicate at baseline (prior to treatment).

### 2.4. Statistical Analysis

Statistical analysis was performed using SPSS software (version 21.0, IBM Corp., Armonk, NY, USA). Continuous variables are presented as mean ± SEM. For normally distributed variables, differences between groups were assessed using Student’s *t*-test, while non-normally distributed variables were analyzed using the Mann–Whitney U test. Comparisons among hypothyroid, hyperthyroid, and control groups were performed using one-way ANOVA for normally distributed data or the Kruskal–Wallis test for non-normally distributed data. Categorical variables were analyzed with the chi-square test. *p*-values < 0.05 were considered significant.

Correlation analysis between bone markers (DKK-1, SOST, OPG), thyroid hormones (FT3, FT4, TSH), and thyroid autoantibodies (TgAb, TPOAb) was performed using Pearson’s correlation coefficient (r). Correlation strength was interpreted according to commonly accepted criteria, as weak (r < 0.4), moderate (r = 0.4–0.6), and strong (r > 0.6), based on standard statistical conventions described in the literature.

## 3. Results

### 3.1. Baseline Characteristics of the Study Population

The clinical and demographic characteristics of the study population are presented in Table 1. To provide an overall comparison between patients and controls, women with thyroid dysfunction were initially analyzed as a single group. There were no statistically significant differences in mean age or body mass index (BMI) between patients and controls (*p* > 0.05). When analyzed separately, hypothyroid and hyperthyroid groups showed comparable age and BMI values, with no statistically significant differences between subgroups or in comparison with controls. The distribution of menopausal status was also similar across all groups, with approximately 60% premenopausal and 40% postmenopausal participants. All patients included in the study had overt thyroid dysfunction at baseline, as defined in the Section 2.

### 3.2. Thyroid Hormones and Autoantibodies

Serum thyroid hormone levels and autoantibody profiles are summarized in Table 2. Both hypothyroid and hyperthyroid women demonstrated significantly altered levels of TSH, FT4, FT3, TPOAb, TgAb, and TRAb compared with controls (*p* < 0.001), confirming the presence of overt autoimmune thyroid dysfunction in the study groups.

TRAb was assessed to further characterize patients with suspected Graves’ disease; however, subsequent analyses of bone-related markers were performed according to thyroid functional status (hypothyroid vs. hyperthyroid), without additional stratification based on antibody status.

### 3.3. Serum Wnt Inhibitors and OPG

**Figure 1:**
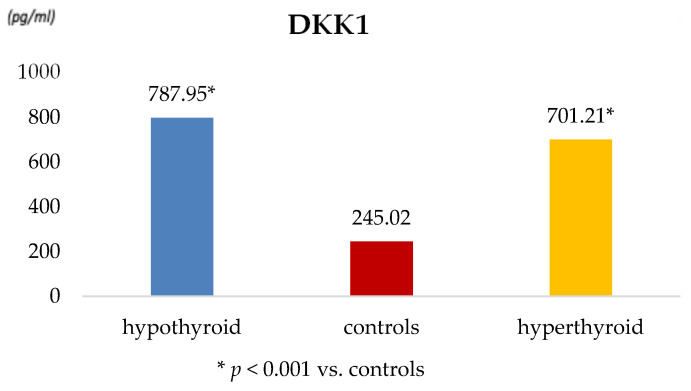
Serum DKK-1 concentrations in hypothyroid and hyperthyroid women compared with controls.

**Figure 2:**
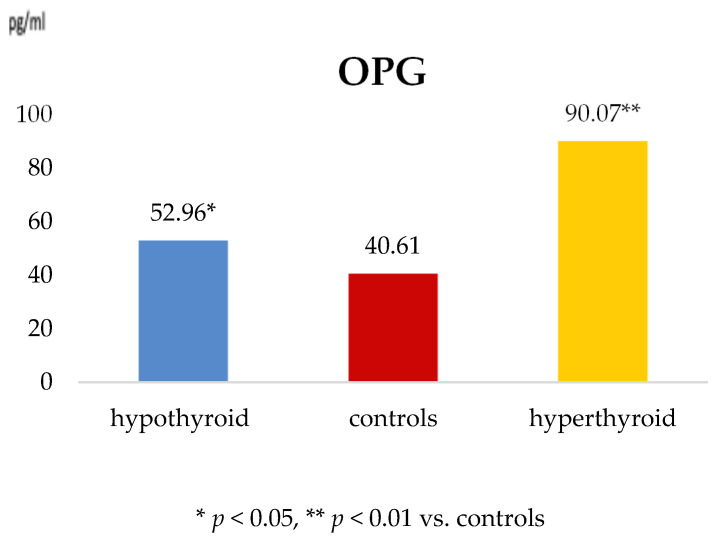
Serum OPG concentrations in hypothyroid and hyperthyroid women compared with controls.

Serum concentrations of DKK-1, SOST, and OPG are presented in Table 3 and illustrated in Figure 1 and Figure 2.

DKK-1 levels were significantly higher in both hypothyroid and hyperthyroid women compared with controls (*p* = 0.001), with a consistent pattern across both patient groups (Figure 1). Sclerostin concentrations were elevated in both hypothyroid and hyperthyroid patients relative to controls; however, these differences did not reach statistical significance. Serum OPG levels were significantly increased in hyperthyroid women and moderately elevated in hypothyroid patients compared with controls (Figure 2), indicating differential regulation of bone resorption pathways depending on thyroid functional status.

### 3.4. Correlation Analysis

Correlation analysis revealed significant associations between selected bone markers and thyroid-related parameters (Table 4).

**Figure 3:**
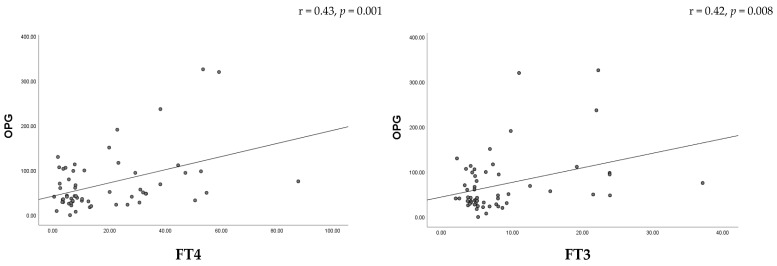
Scatter plot, showing correlation between OPG and thyroid hormones in patients with thyroid dysfunction.

**Figure 4:**
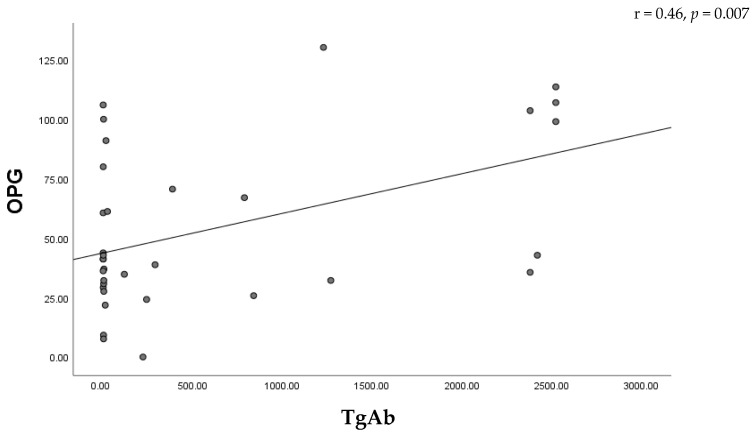
Correlation between OPG and TgAb in hypothyroid patients.

**Figure 5:**
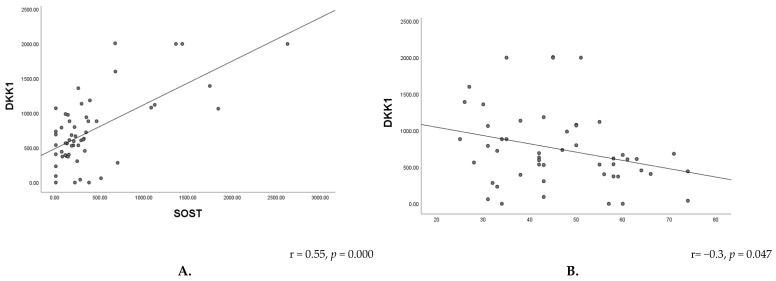
(**A**) Correlations between serum DKK-1 and SOST; (**B**) Correlation between DKK-1 and age in women with overt thyroid dysfunction.

Correlation analysis revealed a moderate positive association between serum OPG levels and thyroid hormone concentrations in women with thyroid dysfunction, including FT3 (r = 0.42, *p* = 0.008) and FT4 (r = 0.43, *p* = 0.001) (Figure 3). In the hypothyroid group, a moderate positive correlation was observed between OPG and TgAb levels (r = 0.46, *p* = 0.007), suggesting a relationship between bone metabolism and thyroid autoimmunity (Figure 4). A strong positive correlation was found between SOST and DKK-1 levels (*p* < 0.001), suggesting coordinated regulation of Wnt signaling inhibitors. In contrast, DKK-1 levels showed a negative correlation with age (*p* < 0.05) (Figure 5). No significant associations were observed between SOST, DKK-1, or OPG and other thyroid function parameters.

## 4. Discussion

The present study investigated the relationship between thyroid dysfunction and bone metabolism in women with newly diagnosed overt thyroid disease, focusing on circulating Wnt signaling inhibitors and OPG. Our findings show significant alterations in DKK-1 and OPG levels, along with specific associations with thyroid hormones and autoantibodies, suggesting that both hormonal and immune-mediated mechanisms may be involved in skeletal changes in thyroid disorders. These mechanisms may involve direct effects of thyroid hormones on bone cells, as well as modulation of bone remodeling through immune pathways and cytokine signaling [1,12,16]. In addition, the observed correlations between DKK-1, SOST, and age highlight the complex regulation of bone remodeling in this setting. These results provide further insight into the pathophysiological links between thyroid function and bone metabolism. When analyzed according to thyroid functional status, distinct patterns of bone metabolism were observed in hypothyroid and hyperthyroid patients.

### 4.1. Hypothyroidism

In women with newly diagnosed hypothyroidism, serum DKK-1 levels were significantly elevated compared with controls, suggesting a potential inhibition of Wnt-mediated osteoblast activity in overt thyroid dysfunction [7,14]. Sclerostin levels were not significantly different, but showed a strong positive correlation with DKK-1, suggesting coordinated regulation of Wnt inhibitors by osteocytes [8,17].

Our findings of elevated DKK-1 levels are in line with previous studies suggesting suppression of Wnt signaling in conditions associated with reduced bone formation [7,14]. However, data on circulating Wnt inhibitors in hypothyroidism remain limited and somewhat inconsistent, likely due to differences in study populations, disease severity, and methodological approaches.

Hypothyroidism is generally associated with reduced bone turnover, characterized by decreased osteoblast activity and a prolonged bone remodeling cycle, while osteoclastic resorption is relatively less affected [1,18]. In addition to thyroid hormone deficiency, elevated TSH levels in hypothyroidism may directly influence bone metabolism. TSH has been shown to inhibit osteoclastogenesis and reduce bone turnover, potentially contributing to the low bone turnover state observed in hypothyroid patients. Although direct evidence linking TSH to circulating Wnt inhibitors is limited, it is plausible that TSH may indirectly modulate these pathways through its effects on bone cell activity [19].

In the present study, serum OPG correlated positively with TgAb levels, highlighting a potential link between thyroid autoimmunity and bone metabolism. This finding suggests that immune-mediated mechanisms may be involved in the regulation of bone resorption through the RANK/RANKL/OPG pathway in autoimmune thyroid disease [12,16,20].

Taken together, these findings suggest that in hypothyroidism, elevated DKK-1 and its association with SOST may reflect reduced bone formation, while increased OPG in relation to thyroid autoantibodies may indicate an additional layer of immune-mediated modulation of bone remodeling. These alterations may represent early biochemical changes in skeletal metabolism prior to measurable reductions in bone mineral density.

### 4.2. Hyperthyroidism

In hyperthyroid women, we also observed elevated serum DKK-1 levels, whereas SOST remained unchanged compared with controls. The positive correlation between DKK-1 and SOST seen in hypothyroid patients persisted, suggesting that coordinated inhibition of Wnt signaling and consequent suppression of osteoblast activity may be a general feature of thyroid dysfunction.

These findings are partially consistent with previous studies indicating altered Wnt signaling in conditions of increased bone turnover; however, data on circulating Wnt inhibitors in hyperthyroidism remain limited and heterogeneous [15,21,22].

Overt hyperthyroidism is known to accelerate bone turnover by directly stimulating osteoclastic activity through elevated thyroid hormones (FT3 and FT4) [1,23]. Thyroid hormones increase RANKL expression in osteoblasts and stromal cells, enhancing osteoclastogenesis and bone resorption [24]. This imbalance, favoring resorption over formation, leads to decreased bone mineral density and increased fracture risk in clinical and subclinical hyperthyroidism [25].

Serum OPG concentrations were elevated and positively correlated with FT3 and FT4 levels, suggesting a compensatory feedback mechanism in response to increased bone resorption. This observation is in line with the established role of the RANK/RANKL/OPG system in regulating osteoclastogenesis and maintaining skeletal homeostasis [10,11].

In addition, experimental studies indicate that excess thyroid hormones modulate the Wnt/β-catenin pathway in bone cells, leading to downregulation of anabolic signals and further inhibition of osteoblast differentiation [26,27]. The elevated DKK-1 levels in hyperthyroid patients may represent a potential additional mechanism by which bone formation is suppressed, beyond the direct catabolic effects of thyroid hormones.

Taken together, these findings are consistent with the concept that in hyperthyroidism, DKK-1 and SOST may reflect reduced osteoblast-mediated bone formation, while OPG elevation represents a compensatory counterbalance to accelerated bone resorption. Monitoring these markers may provide clinically relevant insight into skeletal risk and altered bone metabolism before detectable decreases in bone mineral density occur.

Overall, these findings provide additional insight into the complex interaction between thyroid function and bone metabolism. Elevated DKK-1 levels observed in both hypo- and hyperthyroid patients may be associated with alterations in bone turnover, potentially through effects on osteoblast function. In hyperthyroidism, increased DKK-1 may partly explain the inability of bone formation to compensate for accelerated bone resorption, whereas in hypothyroidism, higher DKK-1 levels may reflect reduced bone turnover and suppressed osteoblast activity. These findings suggest that modulation of Wnt signaling by thyroid hormones may be involved in the observed alterations in bone metabolism in thyroid dysfunction. However, the available data on circulating DKK-1 in thyroid dysfunction are heterogeneous, and the underlying mechanisms remain incompletely understood.

Across all patients, DKK-1 showed a negative correlation with age, suggesting age-related modulation of Wnt signaling. The strong positive association between DKK-1 and SOST further supports coordinated regulation of osteocyte-derived inhibitors in bone remodeling.

From a clinical perspective, combined assessment of DKK-1, SOST, and OPG may serve as a non-invasive approach to detect early biochemical changes in bone metabolism in women with newly diagnosed overt thyroid disease. Although these biomarkers cannot replace direct bone mineral density measurements, they may contribute to early risk stratification and guide clinical monitoring.

### 4.3. Limitations and Future Directions

Limitations of the present study include the relatively small sample size for DKK-1, SOST, and OPG. The absence of direct bone mineral density (BMD) measurements limits the ability to establish the clinical relevance of these biomarkers in relation to skeletal status. In addition, the cross-sectional design precludes causal inferences. The lack of stratification according to menopausal status may also represent a limitation, given the known influence of menopause on bone metabolism. Although the distribution of pre- and postmenopausal women was comparable between groups, residual confounding cannot be fully excluded. Furthermore, the relatively small sample size precluded subgroup analyses, which may limit the generalizability of the findings. Future longitudinal studies incorporating DXA or other bone density assessments are warranted to confirm these associations and to evaluate the predictive value of these biomarkers for bone loss and fracture risk in thyroid disorders [12,13,15].

Overall, our findings suggest that monitoring of DKK-1, SOST, and OPG may provide additional insight into early biochemical changes in bone metabolism in overt thyroid disease; however, their clinical utility requires further validation.

## 5. Conclusions

In conclusion, women with newly diagnosed overt thyroid dysfunction exhibit significant alterations in circulating Wnt signaling inhibitors and osteoprotegerin, reflecting changes in bone metabolism. Elevated DKK-1 levels in both hypothyroid and hyperthyroid patients suggest inhibition of osteoblast-mediated bone formation, while the positive correlation between DKK-1 and SOST indicates coordinated regulation of Wnt pathway activity.

In hyperthyroidism, increased OPG levels and their association with thyroid hormones point to a compensatory response to enhanced bone resorption, whereas in hypothyroidism, the correlation between OPG and TgAb highlights the role of autoimmune mechanisms in skeletal regulation.

Overall, the combined assessment of DKK-1, SOST, and OPG provides insight into early biochemical changes in bone metabolism in overt thyroid dysfunction and may contribute to improved evaluation of skeletal involvement in these patients. Further studies are needed to determine their potential role in predicting bone loss and fracture risk.

## Figures and Tables

**Table 1:** Baseline characteristics of patients and controls.

Parameter	Thyroid Dysfunction (*n* = 62)	Controls (*n* = 33)	*p*-Value
Age (years), mean ± SEM	47.0 ± 1.1	44.6 ± 1.6	>0.05
BMI (kg/m^2^), mean ± SEM	26.7 ± 0.47	26.8 ± 0.71	>0.05
Premenopausal, n (%)	37 (60.4%)	20 (60.4%)	>0.05
Postmenopausal, n (%)	25 (39.6%)	13 (39.6%)	>0.05

Data are presented as mean ± SEM.

**Table 2:** Hormonal and immunological parameters in the study groups.

Parameter	Hypothyroid (*n* = 35) Mean ± SEM	Controls (*n* = 33) Mean ± SEM	Hyperthyroid (*n* = 27) Mean ± SEM
TSH (mIU/L)	46.16 ± 6.36 *	2.04 ± 0.13	0.01 ± 0.00 *
FT4 (pmol/L)	6.68 ± 0.47 *	11.31 ± 0.29	36.00 ± 2.45 *
FT3 (pmol/L)	4.41 ± 0.16 *	5.05 ± 0.12	14.63 ± 1.17 *
TPOAb (IU/mL)	590.82 ± 58.27 *	3.38 ± 1.40	232.21 ± 40.36 *
TgAb (IU/mL)	471.52 ± 115.14 *	0.58 ± 0.12	44.71 ± 16.52 *
TRAb (IU/L)	2.11 ± 1.45 *	0.6 ± 0.11	12.15± 1.75 *

Data are presented as mean ± SEM, * *p* < 0.01 vs. controls. Abbreviations: TSH—thyroid-stimulating hormone; FT4—free thyroxine; FT3—free triiodothyronine; TPOAb—thyroid peroxidase antibodies; TgAb—thyroglobulin antibodies; TRAb—thyrotropin receptor antibodies.

**Table 3:** Serum concentrations of DKK-1, Sclerostin, and Osteoprotegerin.

Parameter	Hypothyroid (*n* = 35) Mean ± SEM	(*p*)	Controls (*n* = 33) Mean ± SEM	(*p*)	Hyperthyroid (*n* = 27) Mean ± SEM
Dkk-1 (pg/mL)	787.95 ± 107.27	0.001	245.02 ± 74.13	0.001	701.21 ± 88.28
SOST (pg/mL)	488.72 ± 123.02	0.064	235.24 ± 49.29	0.570	302.24 ± 66.93
OPG (pg/mL)	52.96 ± 5.76	0.041	40.61 ± 6.12	0.005	90.07 ± 16.54

Data are presented as mean ± SEM. Statistical comparisons were made versus controls. Statistical significance was defined as *p* < 0.05, *p* < 0.01, *p* < 0.001). DKK-1—Dickkopf-1; SOST—Sclerostin; OPG—osteoprotegerin.

**Table 4:** Correlation analysis of bone-related markers and thyroid parameters.

Parameter	r	*p*
OPG-FT3	0.42	0.008
OPG-FT4	0.43	0.001
OPG-TgAb	0.46	0.007
DKK1-SOST	0.55	0.001
DKK1-Age	−0.30	0.047

Correlation coefficients (r) and *p*-values are shown. Pearson correlation analysis was used. Statistically significant correlations were defined as *p* < 0.05. OPG—osteoprotegerin; FT3—free triiodothyronine; FT4—free thyroxine; TgAb—thyroglobulin antibodies; DKK1—Dickkopf-1. SOST—Sclerostin.

## Data Availability

The original contributions presented in this study are included in the article. Further inquiries can be directed to the corresponding author.

## Abbreviations

The following abbreviations are used in this manuscript:

DKK-1—Dickkopf-1; SOST—Sclerostin; OPG—osteoprotegerin; FT3—free triiodothyronine; FT4—free thyroxine; TSH—thyroid-stimulating hormone; TgAb—thyroglobulin antibodies; TPOAb—thyroid peroxidase antibodies. TRAb—thyrotropin receptor antibodies. AITD—Autoimmune thyroid diseases. BMI—body mass index, BMD—bone mineral density, DXA—Dual-Energy X-ray Absorbtiometry
